# Implementing a social prescribing program in primary care units in Portugal: A qualitative study exploring enablers, barriers and lessons learned from the perspectives of stakeholders involved in the program implementation

**DOI:** 10.1371/journal.pone.0306404

**Published:** 2024-06-28

**Authors:** Louíse Viecili Hoffmeister, Ana Gama, Barbara Gonçalves, Cristiano Figueiredo, João V. Cordeiro, Marie Polley, Gisela Souto de Moura, Sónia Dias

**Affiliations:** 1 NOVA National School of Public Health, Public Health Research Centre, CHRC, REAL, NOVA University Lisbon, Lisbon, Portugal; 2 Baixa Family Health Unit, São José Local Health Unit, National Health Service, Lisbon, Portugal; 3 CICS, NOVA Interdisciplinary Center of Social Sciences, Universidade NOVA de Lisboa, Lisbon, Portugal; 4 Meaningful Measures Ltd., Bristol, United Kingdom; 5 School of Nursing, Federal do Rio Grande do Sul University, Porto Alegre, Brazil; University of Coimbra: Universidade de Coimbra, PORTUGAL

## Abstract

Social prescribing (SP) is a promising intersectoral strategy of integrated and person-centered care that can improve individual health and well-being by going beyond medical treatment, potentially reducing overall disease burden on health system. SP addresses health and social determinants of health by linking patients to community responses, i.e. services and initiatives fostering social interaction, physical activity and creativity, among other health-promoting aspects, provided by local public, private, and non-profit entities. There is limited research on the implementation processes of SP beyond the UK, hindering improvement and scale-up. This study aims to identify enablers and barriers of implementation of an SP program conducted in Portugal. A participatory and qualitative approach was used to assess the implementation of an SP program in health units. Semi-structured interviews were conducted with family doctors, social workers and representatives of community partners who participated in SP implementation. The Consolidated Framework for Implementation Research was used to conduct thematic analysis. The main enablers of SP implementation included its recognition as an evolution towards a holistic model of health, the personal characteristics of professionals as being proactive, motivated, and concerned with social determinants of health, and the communication strategy used to engage the stakeholders. Perceived challenges included raising users’ awareness of SP and ensuring intervention adherence. Lack of preparedness for intersectoral working processes, including insufficient communication channels, limited community responses and the need for a more systematic collection of data on activities adherence and progress were also highlighted as barriers to SP implementation. SP implementation seems simple, but the results show that in practice, we are facing a complex intervention with multiple stakeholders, diverse community responses and factors influencing project success. A deeper understanding of SP specificities, local context, enablers, and barriers is vital to develop strategies for improvement and successful implementation, ensuring scalability and sustainability.

## Introduction

Addressing the social determinants of health and reducing health inequities is a major challenge for health services worldwide, especially for Primary Health Care (PHC). Individuals from lower socioeconomic status tend to have fewer opportunities and resources to adopt healthy lifestyles [1,2]. In addition, social inequalities often translate into disparities in health care access, leading to increased disease burden, higher mortality rates and decreased life expectancy [3–5]. Furthermore, social isolation and loneliness are risk factors for all causes of mortality and result in an increased demand for PHC services [6–10].

Clinical approaches based solely on disease treatment and the biomedical model, have shown to be insufficient to comprehensively meet patients’ needs [11,12]. To achieve better health and well-being, individuals must have their social, economic, and emotional issues addressed [3]. In this regard, complex interventions have been designed and implemented with the purpose of providing holistic and comprehensive care that addresses all dimensions of human well-being [13]. SP is one of the well-known practical models of complex intervention that focuses on addressing individual social, economic and emotional needs, within an integrated and person-centred care approach [14].

This integrative model strengthens the network between the health and social sectors and is operationalized mainly through the patient’s referral process and the collaboration between a healthcare provider and a link worker or community navigator [14]. Link workers carry out detailed assessments of the patient’s needs and an individual care plan is co-created. This plan outlines how to support a person’s needs by using services in the community where the individual lives, considering the specificities of the existing resources [14]. These services are related to e.g. physical activity, language classes, artistic and cultural activities, gardening, financial services, food and housing support, and intend to respond to non-medical needs, such as social isolation, mental health issues, financial difficulties, migrant integration, or functional dependency, among others.

It is important to evaluate the processes of implementation of complex interventions such as SP, including its enablers and barriers, to understand what works and does not work, for whom, and in which circumstances [15]. In particular, understanding the drivers of implementation success and failure related to the intervention context is crucial to inform improvement and design of scalable and sustainable interventions [16,17]. In order to assess the key determinants of the implementation of SP projects it is fundamental to use adequate theoretical frameworks [18]. Several models have been developed to provide a structure for describing the process of putting effective interventions into practice, as well as a subsidy to analyse which factors influence implementation efforts and outcomes [18,19]. The Consolidate Framework for Implementation Research (CFIR) is a key theoretically grounded framework, which serves to systematically guide the assessment of implementation contexts and the identification of multilevel factors influencing the implementation outcomes of an intervention [20].

Using the CFIR, our study aimed to identify enablers and barriers to the implementation of an SP program in Lisbon from the perspectives of stakeholders involved in the program implementation.

## Methods

### Setting

The SP program was implemented in two Family Health Units (FHUs) in Lisbon, which provide general health care to approximately 29.000 people of all ages. Users of these units include people living in socially and economically vulnerable situations, such as elderly in isolation, recently arrived migrants, unemployed people, many who have low levels of education, and people experiencing homelessness [21]. These FHUs are staffed with family doctors, nurses and clinical secretaries and receive specialized support from social workers, psychologists, physiotherapists, and occupational therapists [22].

**SP program.** The SP program has been implemented in these FHUs with the aim of responding to users’ socioeconomic and emotional needs through the community assets available, to improve their health, well-being, and quality of life. The project also aims to improve communication and collaboration between health professionals, social workers and community partners operating in the territories covered.

FHUs’ patients of all age groups and with any non-clinical needs are referred to the SP program by their healthcare provider, or voluntarily enter the program. Clinical secretaries also assist in proactive referral to the SP program during their front-desk contacts.

During regular appointments, whenever healthcare providers identify non-clinical needs, they explain the SP program to their patients and ask about their interest in taking part. Upon patient consent, healthcare providers complete a referral form on a dedicated electronic platform, which sends an automatic email to the FHU’s social worker and other involved healthcare providers (e.g., family doctor and family nurse) with the patients’ referral information (e.g. reasons for referral, presence of chronic disease, need for a translator). Patients are then schedule an SP appointment with the social worker. Follow-up of patients who do not schedule this appointment within 2–3 weeks is guaranteed by a clinical secretary.

In this SP program, social workers have taken on the role of SP link workers. In the first SP appointment, the social worker assesses the patient’ needs, their preferences and motivations, and jointly they devise a personalised action plan. The social worker introduces the patient to activities provided by the FHU, such as walking groups and health literacy sessions, and activities offered by partners of the social/voluntary sector in the community.

Following patient’ agreement regarding the most suitable strategies to address the identified individual needs, the social worker refers the patient (by e-mail or telephone) to the appropriate services in the community. The SP program also includes patient follow-up after the referral process, to assess patient’s pathway and achieved outcomes and, if necessary, to schedule new appointments. Throughout the SP program, the social worker updates and shares information with the healthcare provider. For this purpose, instruments were developed and adapted to expedite communication between different stakeholders. The email with referral information could be used to case discussions between social worker and healthcare providers (family doctor, family nurse and professional who made the referral) and for the social worker to give feedback on the patient’s progress. The participation in the SP program is considered complete when the patient has their needs met. If a patient does not attend the scheduled appointments or drops out of agreed activities, then their participation is also ended.

In the planning phase of the program, the implementation team adopted a participatory and collaborative approach to plan and develop tools and processes. Stakeholders were invited to participate in the discussions and decisions related to the dedicated platform for referrals and other communication tools, and to help adapt the SP model to the Portuguese context. A key strategy used by the implementation team to engage all stakeholders in SP implementation was to reinforce the communication through monthly meetings with community partners, monthly feedbacks to the health unit teams and frequent publication of newsletters. Specifically, regarding the organization of meetings, different strategies were used for promoting collective participation, including clinical cases discussion, workshops, and holding meetings in different locations.

**Fig 1:**
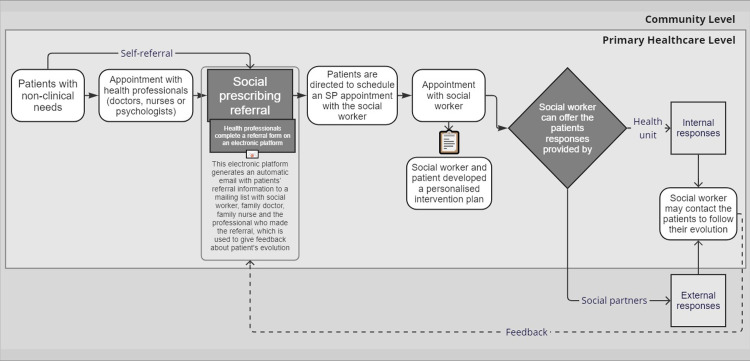
Circuit implemented in the SP program in Lisbon.

Overall, the implementation of the SP program involved approximately 20 professionals, from executive directors and health unit coordinators to family doctors, social workers and focal points of community partners. Professionals from the FHUs who were involved in SP implementation redirected approximately four hours per week of their time to develop the program’s activities. No additional funding was provided to run the program. Fig 1 illustrates the intervention strategy.

### Study design and population

This study is part of a large evaluation research on the SP program, comprising quantitative and qualitative components of data collection on the perspectives of diverse stakeholders. During the implementation phase (first three years), approximately 700 patients were referred to the SP program.

Within this participatory research project, a qualitative study was conducted to assess the implementation of the SP program in Lisbon. The study consisted of online individual semi-structured interviews with stakeholders who participated in the SP implementation process, to capture the experiences of those implementing the intervention, including perceived enablers and difficulties [23].

The study population comprised family doctors, social workers and representatives of community partners involved in the SP implementation. Professionals from the FHUs were invited to participate in the regular meetings between the health and the evaluation research teams. Community partners were invited to participate by email. Recruitment of participants continued until saturation was achieved. The recruitment period started on 1st August 2020 and ended on 30th July 2021. This manuscript adheres to the Standards for Reporting Qualitative Research (SRQR) [24].

### Data collection

The interviews were scheduled according to the participants preferences. At the beginning of each interview, participants were informed about the study’s objectives and ethical assumptions (voluntary participation, anonymity, confidentiality of data). Verbal informed consent was obtained by all study participants and documented in audio records. The interviews were conducted by video conference and lasted on average 45 minutes. A semi-structured guide was used to conduct the interview, covering the interviewees’ perceptions and experiences related to the implementation of SP program, including motivation for implementation, implementation steps, enablers and barriers. The interviews were anonymized, audio recorded, transcribed verbatim. After the interviews, the recordings were transferred onto an encrypted file with secured access and deleted from the audio recorder.

This study was approved by the Ethics Committee for Health of the Regional Health Administration of Lisbon and Tagus Valley (reference 5 2020/CES/2020).

### Data analysis

Thematic analysis was conducted according to the six steps outlined by Clarke & Braun [25]: I) Familiarising with data; II) Generating initial codes; III) Searching for themes; IV) Reviewing themes; V) Defining and naming themes; VI) Producing the report. A deductive approach was applied, in which the CFIR was used as a framework to orientate the thematic analysis and the codes identified in the data were grouped in according to existing domains and constructs derived from the CFIR. In this analysis, the themes were presented as domains and categories were described as constructs according to CFIR nomenclature [20]. The CFIR is composed of five major domains divided into 39 constructs (Table 1), each of them possibly influencing an intervention’s implementation as a facilitator or a barrier to the implementation of the SP program [20].

**Table 1:** Description of CFIR domains and constructs [20].

Construct	Construct Description
**Domain: Intervention characteristics** **Description: Characteristics of the intervention being implemented in a particular organization**	
Intervention Source	Perception of key stakeholders about whether the intervention is externally or internally developed.
Evidence Strength & Quality	Stakeholders’ perceptions of the quality and validity of evidence supporting the belief that the intervention will have desired outcomes.
Relative Advantage	Stakeholders’ perception of the advantage of implementing the intervention versus an alternative solution.
Adaptability	The degree to which an intervention can be adapted, tailored, refined, or reinvented to meet local needs.
Trialability	The ability to test the intervention on a small scale in the organization, and to be able to reverse course (undo implementation) if warranted.
Complexity	Perceived difficulty of implementation, reflected by duration, scope, radicalness, disruptiveness, centrality, and intricacy and number of steps required to implement.
Design Quality & Packaging	Perceived excellence in how the intervention is bundled, presented, and assembled.
Cost	Costs of the intervention and costs associated with implementing the intervention including investment, supply, and opportunity costs.
**Domain: Outer setting** **Description:** Economic, political, and social context in which an organization resides	
Patient Needs & Resources	The extent to which patient needs, as well as barriers and enablers to meet those needs, are accurately known and prioritized by the organization.
Cosmopolitanism	The degree to which an organization is networked with other external organizations.
Peer Pressure	Mimetic or competitive pressure to implement an intervention; typically because most or other key peer or competing organizations have already implemented or are in a bid for a competitive edge.
External Policy & Incentives	A broad construct that includes external strategies to spread interventions, including policy and regulations (governmental or other central entity), external mandates, recommendations and guidelines, pay-for-performance, collaboratives, and public or benchmark reporting.
**Domain: Inner setting** **Description:** Features of structural, political, and cultural contexts in which the implementation process will proceed	
Structural Characteristics	The social architecture, age, maturity, and size of an organization.
Networks & Communications	The nature and quality of webs of social networks and the nature and quality of formal and informal communications within an organization.
Culture	Norms, values, and basic assumptions of a given organization.
Implementation Climate	The absorptive capacity for change, shared receptivity of involved individuals to an intervention, and the extent to which use of that intervention will be rewarded, supported, and expected within their organization.
Tension for Change	The degree to which stakeholders perceive the current situation as intolerable or needing change.
Compatibility	The degree of tangible fit between meaning and values attached to the intervention by involved individuals, how those align with individuals’ own norms, values, and perceived risks and needs, and how the intervention fits with existing workflows and systems.
Relative Priority	Individuals’ shared perception of the importance of the implementation within the organization.
Organizational Incentives & Rewards	Extrinsic incentives such as goal-sharing awards, performance reviews, promotions, and raises in salary, and less tangible incentives such as increased stature or respect.
Goals and Feedback	The degree to which goals are clearly communicated, acted upon, and fed back to staff, and alignment of that feedback with goals.
Learning Climate	A climate in which: a) leaders express their own fallibility and need for team members’ assistance and input; b) team members feel that they are essential, valued, and knowledgeable partners in the change process; c) individuals feel psychologically safe to try new methods; and d) there is sufficient time and space for reflective thinking and evaluation.
Readiness for Implementation	Tangible and immediate indicators of organizational commitment to its decision to implement an intervention.
Leadership Engagement	Commitment, involvement, and accountability of leaders and managers with the implementation.
Available Resources	The level of resources dedicated for implementation and on-going operations, including money, training, education, physical space, and time.
Access to Knowledge & Information	Ease of access to digestible information and knowledge about the intervention and how to incorporate it into work tasks.
**Domain: Characteristics of individuals** **Description:** Individuals involved with the intervention and/or the implementation process. Individuals can be the representatives of cultural, organisational, professional, and personal mindsets, norms, interests, and affiliations	
Knowledge & Beliefs about the Intervention	Individuals’ attitudes toward and value placed on the intervention as well as familiarity with facts, truths, and principles related to the intervention.
Self-efficacy	Individual belief in their own capabilities to execute courses of action to achieve implementation goals.
Individual Stage of Change	Characterization of the phase an individual is in, as he or she progresses toward skilled, enthusiastic, and sustained use of the intervention.
Individual Identification with Organization	A broad construct related to how individuals perceive the organization, and their relationship and degree of commitment with that organization.
Other Personal Attributes	A broad construct to include other personal traits such as tolerance of ambiguity, intellectual ability, motivation, values, competence, capacity, and learning style.
**Domain: Implementation process** **Description:** An interrelated series of sub-processes that do not necessarily occur sequentially, but ideally need to be all aligned in the same general direction: effective implementation	
Planning	The degree to which a scheme or method of behaviour and tasks for implementing an intervention are developed in advance, and the quality of those schemes or methods.
Engaging	Attracting and involving appropriate individuals in the implementation and use of the intervention through a combined strategy of social marketing, education, role modelling, training, and other similar activities.
Opinion Leaders	Individuals in an organization who have formal or informal influence on the attitudes and beliefs of their colleagues with respect to implementing the intervention.
Formally Appointed Internal Implementation Leaders	Individuals from within the organization who have been formally appointed with responsibility for implementing an intervention as coordinator, project manager, team leader, or other similar role.
Champions	“Individuals who dedicate themselves to supporting, marketing, and ‘driving through’ an [implementation]” [101] (p. 182), overcoming indifference or resistance that the intervention may provoke in an organization.
External Change Agents	Individuals who are affiliated with an outside entity who formally influence or facilitate intervention decisions in a desirable direction.
Executing	Carrying out or accomplishing the implementation according to plan.
Reflecting & Evaluating	Quantitative and qualitative feedback about the progress and quality of implementation accompanied with regular personal and team debriefing about progress and experience.

## Results

For this study, a total of 15 participants were invited to participate, resulting in nine participants enrolled in the study, including two health professional who make referral to SP, two link workers and five focal points of community partners (from local authorities, local associations, and non-governmental organizations). The majority (n = 8) of participants were female, 5 aged between 30–40 years, 5 were social workers and 2 family doctors. Overall, 5 domains and 9 constructs emerged from the interviews narratives referring to both enablers and barriers to the implementation of the SP program (Table 2).

**Table 2:** Enablers and barriers according to CFIR.

CFIR Construct	Enablers	Barriers
**CFIR Domain: Intervention characteristics**		
Relative Advantage	Adoption of the holistic model of health	
	More agile and effective intersectoral action	
Adaptability	Tools constructed and adapted to the local context	
**CFIR Domain: Outer Setting**		
Needs & Resources of Those Served by the Organization	The perception that SP addresses the population needs	Lack of responses in specific areas and for specific populations
		Lack of patients’ awareness about the benefits of the SP program
**CFIR Domain: Inner Setting**		
Structural Characteristics	Proactive, motivated teams, concerned about the influence of social determinants of health	Rigid working processes and structure of the social service in PHC
	Social workers integrated and physically present in the health units	
Readiness for Implementation	Availability of different resources (e.g. the existence of a social service in the health unit; a timeframe set to dedicate to the program; support of external partners)	Lack of resources (e.g. funding for both health and community services dedicate to SP, reduced number of human resources and time available, lack of well-developed intersectoral communication channels)
		Insufficient community responses available
**CFIR Domain: Characteristics of Individuals**		
Knowledge & Beliefs about the Innovation	Perception of stakeholders involved in SP that the intervention is important, adds value and brings different benefits	
**CFIR Domain: Implementation Process**		
Engaging	Communication strategy used to engage the stakeholders involved (e.g. frequent communication between health and social actors, dynamic meetings)	Reduced patients’ adherence
	Engagement of key actors (e.g. leaders of community partners, experts in implementing SP)	
Executing	Increased networking and articulation between health and social sectors	Limited responsiveness of the services involved
Reflecting & Evaluating	Close monitoring of activities and sharing outcomes with partners	Lack of information regarding the progress of activities after referral

### Enablers

Overall, there were 9 constructs identified as enablers to the SP implementation when analysing the interviews using the CFIR, and these enablers were present in every CFIR domain (see Table 2). These will be explained in more detail below.

#### Intervention characteristics

According to the interviewees, the SP program is a solution to tackle the social determinants of health. The SP program was seen by the healthcare providers as an evolution towards a holistic model of health, leaving behind the currently favoured biomedical model. The interviewees mentioned that, compared to the traditional health-social articulation praxis, the SP program brought greater proximity between the different participating institutions and a more agile and effective intersectoral articulation:

*“(…] and so*, *we clearly saw this advantage of everything being faster and more efficient*.*” (ID5*, *community partner*, *female*, *social worker)*

According to some interviewees, the SP program seemed flexible enough to be adapted to local context and needs. The fact that the tools (e.g. referral form, flyers, shared files) used for communication between the different stakeholders were tailored to the intervention features and context specificities was highlighted as a facilitator. It was the case of adapting the instruments to include the language preferences, due to the high proportion of migrants among the population served by the program:

*“These instruments have some particularities*, *as the population was very multicultural*, *there was a need to understand if a translator was needed or not*, *there was a need to make a series of specific response fields (…)” (ID1*, *doctor*, *male*, *family doctor)*

#### Outer setting

The interviewees mentioned that the variety of socioeconomic and emotional needs of populations justifies the implementation of an SP program. Healthcare providers and social workers also identified specific issues in the community they served: strong social inequalities, housing and food problems, including migrant populations, elderly in isolation or with dementia, domestic violence, ill-treatment and young people with economic needs. The perception that some of these needs could be met through such an innovative program such as SP was a positive motivating factor to implement the intervention:

*“We had so many elderly people*, *we didn’t realise that we had so many people in situations of isolation and even so many people who were sometimes homeless or even living in very complicated conditions at home*.*” (ID3*, *doctor*, *female*, *family doctor)*

#### Inner setting

Health professionals’ characteristics were frequently reported by interviewees as important enablers of SP implementation. The fact that the teams implementing the SP program in FHUs were proactive and highly motivated, concerned about the influence of social determinants on patients’ health outcomes, was highlighted by interviewees as a relevant facilitator of the intervention implementation:

*“It is a [health] unit in which the professionals are new*, *of a very young age*, *and therefore*, *they have a different awareness*, *from the moment they start to look… when they have a perspective of looking at the patient in various dimensions… therefore it’s a facilitator*.*” (ID2*, *link worker*, *female*, *social worker)*

Furthermore, according to some interviewees, the physical presence of the social worker in the FHU enables more frequent and closer contact with the health professionals also involved in the program, which improves trust and communication:

*“Physically it makes a lot of difference (*…*) everything is easier*, *both for patients and professionals*, *having the social worker in the health centre (*…*)*.*” (ID4*, *link worker*, *female*, *social worker)*

One other aspect also mentioned as an implementation facilitator was the availability of different resources, including having a social service in the health units, and having a specific timeframe set for staff? to dedicate to the program. Moreover, support of external partners, such as provision of a space to hold meetings with the community partners was also identified as beneficial.

#### Characteristics of individuals

A facilitator of the program implementation mentioned by the interviewees related to the positive perceptions of the stakeholders toward the SP program. Interviewees showed great interest in the SP intervention and described it as interesting and an adding value to existing services. Several benefits associated were mentioned, such as the possibility of closer intercommunication with the community, greater visibility of the social worker’s role, the ease in which patients’ needs can be addressed, and the opportunity to fulfil their institution’s mission:

*“(…) through this project we are able to have a closer articulation with the community and*, *on the other hand*, *it also gives greater visibility to the profession of social worker in PHC*.*” (ID2*, *link worker*, *female*, *social worker)*

#### Implementation process

According to the interviewees, the communication strategy used to engage stakeholders in the program implementation encouraged health professionals to get involved. Dynamic and regular meetings strengthened transparency about the project, generating greater proximity and closer ties with the community partners, allowing them to share their experiences.

*“Teamwork*, *meetings*, *I think are very important for us to be able to maintain and strengthen these ties*, *because this is what will then enable us to carry out our work in the community*, *for us to be able to reach people more efficiently and be able to respond to their needs*.*” (ID6*, *community partner*, *female*, *social worker)*

*“In the meetings*, *there is a scientific part*, *which is always an interesting approach for us who are not from the academic area*, *(*…*) there was a discussion of concrete cases on how the SP was working*, *and workshops about the weaknesses*, *the constraints*.*” (ID7*, *community partner*, *female*, *social worker)*

The interviewees also highlighted that the engagement of key actors to the SP program through their participation in the project meetings contributed to the program’s progress, with a positive impact on the public recognition of the program. Those actors included leaders of community partners, i.e. individuals within an organization who have formal or informal influence on the attitudes and beliefs of their colleagues with respect to the intervention, as well as personalities with experience in implementing SP in other countries:

*“(*…*) there were several leadership people*, *people who are respected*, *who listened to the project and found it interesting*, *(*…*) it’s not that it was a very formal support*, *I think this in a way was also a facilitator*.*” (ID1*, *doctor*, *male*, *family doctor)*

Interviewees also felt that, regarding the execution phase, a strong and proximal relation between professionals, as well as the intersectoral work, increased throughout program’s implementation. The networking and articulation between the social and health sectors made the work more dynamic and enabled frequent dialogue between diverse entities:

*“Some social organisations that did not meet before*, *now with the SP they meet regularly*, *and this has added great value*, *(*…*) I think that SP has opened doors to this more facilitated dialogue with other entities (*…*)*.*” (ID7*, *community partner*, *female*, *social worker)*

Finally, interviewees noted that the close monitoring and evaluation of SP referrals and sharing outcomes between health teams and community partners were facilitating factors of SP implementation.

### Barriers

There were 6 CFIR constructs identified as barriers to the SP implementation in the interviews narratives, and these barriers were present in 3 CFIR domains: Outer setting, Inner setting and Implementation Process (see Table 2). These will be explained in more detail below.

#### Outer setting

Despite existing resources in the community, the interviewees described a lack of services and initiatives in specific areas, such as arts, music, tackling sedentary lifestyles, housing and migration-related needs. Interviewees also mentioned a gap in responses available for the population under 65 years of age, especially those related to physical exercise:

*“There are not as many as desired responses related to housing*, *sedentarism*, *and physical exercise*, *and there is an age group for which the responses are all to be paid*, *there is no free access*, *and under 65 years of age we don’t have very affordable responses*.*” (ID2*, *link worker*, *female*, *social worker)*

Patients’ lack of awareness of the SP program itself, including the role of the social workers in improving their health and well-being, was also identified as an implementation barrier. Many interviewees mentioned feelings of mistrust and disbelief among patients when non-traditional solutions to their problems were proposed to them, such as physical activity, participation in art classes, or attendance at a day-care centre. According to the interviewees, patients’ preconceptions and unawareness of the benefits of community responses impacted their receptivity and adherence to the suggested activities within the SP scope, hindering the implementation of the SP program:

*“(…] the patients who are often over users*, *isolated*, *with multiple diseases*, *who often have non-specific problems*. *(*…*) it is precisely these people to whom you are referring a new*, *innovative service*, *and the person naturally goes suspicious*.*” (ID1*, *doctor*, *male*, *family doctor)*

#### Inner setting

The interviewees also mentioned that the rigid working processes and structure of the social service in PHC require social workers to attend patients from two to three different FHUs, which increases workload and reduces the time available for the SP program activities:

*“Sometimes it was time*, *(…) time that we have for other things as well*, *for other functions*, *and sometimes it was a barrier*, *we couldn’t do it in the timing that we wanted*, *because I don’t do just this (…]*.*” (ID2*, *link worker*, *female*, *social worker)*

Interviewees also described the lack of funding for both health and community services to dedicate to SP, the reduced number of human resources, the scarcity of time and the lack of well-developed intersectoral communication channels (e.g. a digital platform) to support articulation between partners regarding SP activities. These were factors that all negatively influenced the progress of the program:

*“Actually*, *it’s a question of organisation and funding*. *Firstly*, *funding (…) to have more users or more social response vacancies we would need to have more human resources*. *Human resources means more money*, *so we would need to have more funding (*…*]*.*” (ID5*, *community partner*, *female*, *social worker)*

Both the healthcare providers and community partners reported the saturation of responses and the lack of vacancies in the activities provided by the community services as factors that negatively influence the SP implementation. The lack of responses to some specific needs caused constraints in patients’ referrals, which according to some social workers, created false expectations in patients:

*“(*…*] I am creating expectations in patients when I say that I can help*, *I am creating expectations*. *The problem is that then*, *this resource does not depend on me*, *and those expectations that we are creating in patients are often let down because those who [should] have the answer do not have an answer*.*” (ID4*, *link worker*, *female*, *social worker)*

#### Implementation process

Despite a regular invitation to participate, interviewees highlighted the overall low participation of patients in the SP program as a barrier to the implementation of the program. Limited responsiveness of the health and social services, such as restricted time spare for appointments with social workers, and lack of community responses available, had a negative effect on the program operationalization. These limitations led to a long waiting time for SP appointments and hindered the capacity of social workers to follow up on patients’ progress:

*“(…] Then another barrier is the timing of the response*, *which is not possible (…) it continues to be our time*, *our availability for the project*. *Before the implementation*, *this was not a problem*. *This was after implementation*, *the problem was in the timings of the responses*, *in the existence of responses in the community*.*” (ID2*, *link worker*, *female*, *social worker)*

The lack of data regarding the progress of activities after referral was also mentioned as a barrier that generated frustration among community partners. In particular, a better understanding of the reasons why patients did not engage in SP activities is needed. This could be explored in the evaluation of the SP program in order to address difficulties in engaging patients and find solutions to improve their acceptability and adherence to SP:

*“It is something that you really have to evaluate*, *if people are not actually attending*, *if they miss out*, *and why do they miss out (…]*.*” (ID3*, *doctor*, *female*, *family doctor)*

## Discussion

This study describes the first Social Prescribing project in Portugal, based on evaluation research, enabling us to monitor the implementation process closely and systematically. Conducting an evaluative component in SP projects has been pointed out as a strategy to reduce the evidence gaps in the area [26].

Most evaluation studies on SP focus mainly on the effectiveness of the intervention in terms of patient outcomes, while fewer studies have referred to the implementation process itself [26–28]. Understanding this process from the perspectives of relevant stakeholders allows to identify barriers and enablers, which can help health professionals, managers, policymakers, and other key stakeholders to develop effective projects in this area [28]. Increased knowledge of the SP implementation processes can also assist in the development of improvement strategies [16].

SP has a solid conceptual ground and implementing it seems simple. Still our findings show that, in practice, SP is a complex intervention that involves multiple stakeholders, diverse community responses and is subject to multilevel factors that can highly impact how successful its implementation turns out to be. SP has elements of complexity across several domains: the components and properties of the intervention itself, the implementation processes, the characteristics of the context and the features of the recipient populations [29].

This study allowed to identify several enablers and barriers of implementation of the SP program from the perspective of relevant stakeholders. The holistic approach of SP emerged as a key facilitator for stakeholders’ uptake and engagement in the program. In this study, participants referred to the SP intervention as an opportunity to respond to the non-clinical needs of the population. In fact, SP acknowledges that needs, such as social isolation, mental health issues, lack of physical activity, and poor living conditions, can significantly impact a person’s health condition and well-being [30]. SP is a strategy to put into practice the holistic paradigm of health care and recognizes the importance of addressing the broader determinants of health and well-being beyond traditional medical interventions [31–34]. Rather than solely relying on medication or medical treatment, SP considers the social, emotional, and practical needs of individuals, placing the person at the center of care [33]. The preparation of a personalised action plan within a co-design approach enhances trust between patients and stakeholders and fosters a sense of control, encouraging them to consider non-clinical aspects while actively participating in treatment choices [35]. Accordingly, past studies have reported the importance of adopting a de-medicalization health approach by general practitioners, especially for patients who have social issues as primary causes for their health condition, thus contributing to making the health system more effective and sustainable [32,36]. A facilitating factor of SP implementation that emerged as well was professionals’ positive attitudes toward the intervention, especially the underlying holistic perspective of patients’ health and well-being, and an understanding of the influence of social determinants on health, individual or collective [32–34]. By taking a holistic, patient-centered, approach and considering the individual’s circumstances, SP allows to empower individuals, enhances their social connections, and promotes self-care, thereby improving their overall well-being and promoting more equitable care [33,37]. Furthermore, it enables service users to express their priorities and align their needs and interests with the available resources [38].

Another facilitator described by the participants in our study was the close proximity of the social workers to the community and their physical presence in the Health Units. In fact, a good relationship between the link workers and the other healthcare parties involved in SP has been documented in previous research as important as it facilitates the implementation and delivery of SP programs [16]. Accordingly, staff of integrated care services referred that working together in the same building in a collaborative manner improved the quality and frequency of communication, and the agility in solving patients’ problems [39]. Furthermore, structured and regular contact between link workers and health professionals was shown to serve as a reminder about the SP program, encouraging a higher number of referrals [16].

Effective communication was identified as a facilitator, particularly when frequent and close intersectoral communication is adopted. Such communication between health professionals, link workers, and social partners is essential for the success of SP projects [40], by promoting a more collaborative work environment and improving information sharing about patients’ needs [19,33]. Interpersonal relationships between link workers and different healthcare professionals and collaborative work through e.g. shared learning sessions and team meetings can be a facilitator of implementing SP [41].

Other contextual factors influencing the implementation of the SP program relate to the link workers, such as the number of link workers, their time availability, and the complexity of their role. The link worker plays a central role within an SP program and their competence lies in their ability to effectively engage, exhibit empathy, actively listen, empower, and motivate individuals [42]. According to some interviewees in the present study, the link workers´ high workload for the time available often leads to long waiting times for SP consultations. Consequently, this can increase the risk of non-engagement of patients and hinder the effective performance of SP services [28,33,43,44]. Similarly, the involvement of link workers was observed to be influenced by various factors. Such as, the timing of link workers’ engagement in the planning and execution of SP initiatives, particularly their early involvement, has been observed to enhance their motivation and commitment to the project considerably [45], whereas substantial workloads and time constraints have been identified as the main factors deterring link workers from engaging in SP initiatives [46]. A context of limited resources may lead link workers to respond primarily to immediate and urgent demands, reducing their capacity to develop community involvement initiatives or even to follow up with patients with complex needs [33]. In this sense, it is important to understand the complexity, responsibilities and skills inherent to the link worker role, and set boundaries on what is part of its functions [46,47]. According to Hayes et al. 2023 [48], link workers, particularly those overseeing multiple sites, expressed challenges in their interactions with clinical teams, highlighting a sense of disconnectedness. Social prescribers often find themselves operating separately from the healthcare system, which impedes collaboration, hampers patient contact and receiving referrals [48]. Some link workers feel forgotten and struggle with the process, which ultimately impacts referral flow and desired outcomes [48]. The visible presence of the link worker, the feedback from link workers regarding the progress of people they had referred into the scheme and the closely work with health colleagues are strategies that promote early collaboration and greater involvement in the SP process of allied stakeholder buy-in [49].

Indeed, communication was also identified as a barrier in this study when there was a lack of well-established communication channels. Similarly, in a previous study, a clearly defined channel for conveying information about patients’ needs was not explicitly established; instead, there was a prevailing dependence on informal monitoring of implementation, as opposed to formal mechanisms [41]. To tackle this issue, the implementation of computerized communication strategies, such as IT systems and team meetings, seems to facilitate the communication between stakeholders and enable the discussion of complex cases [32,34].

The readiness of the context to implement SP, including, for instance, the community structure, human resources, and patients’ acceptability, is key to achieving successful implementation [23,50], and the findings of our study demonstrate this. Most interviewees referred that the reduced local community resources currently available was, in fact, a hindering factor to the progress of the program. Namely, they mentioned the limited amount and variety of existing local responses, as well as the lack of funding for the design and development of complementing social responses. Lack of resources has been previously identified a challenge associated with the absence of support for services from regional governing bodies, as well as difficulties in securing grants and alternative sources of funding, along with the recruitment and retention of volunteers [38,51]. As documented elsewhere, limited resources, especially low financing for supporting services, can constitute barriers to engaging community partners [16]. A strong and comprehensive community sector is vital for a successful SP intervention, which requires adequate funding and appropriate infrastructure [52,53]. Simultaneously, the resources in the community need to be fully aligned with patients’ needs and accessible; otherwise, the SP activities may be overcrowded or less responsive [33]. A strong, integrated, and comprehensive voluntary and community sector is vital for a successful SP intervention, which requires adequate funding and appropriate infrastructure [52,53].

In our study, patient adherence was pointed out exclusively as a barrier to implementation. The patient attrition throughout the project was a frequent difficulty referred by the interviewees. According to participants, decreased patient participation occurs through non-booking or missing SP appointments and non-adherence to the proposed activities. Generally, the low engagement of patients is cited as one of the biggest barriers to the implementation and delivery of SP services [16]. Factors affecting patient engagement in SP projects have been identified, including high waiting time, misperception about the health-promoting role of primary health care, misalignment between patient expectations and the SP model of care, lack of interest in SP, skepticism around its potential benefit, and lack of economic support (e.g. for project activities and transportation to SP-related services) [16,17,28]. Strategies to increase patient adherence to SP have been recommended, such as developing activities free of charge, in-person visits, making telephone contacts with first-time referred patients, or even accompanying individuals to their initial session and promoting patients confidence and motivation, while engaging them in health literacy promotion [17,43,54,55]. Offering peer support, peer mentors or buddy systems in understanding health information and interacting with healthcare professionals could also prove advantageous [56].

When introducing SP as a crucial component of patient-centered care, the level and nature of support provided should be tailored to align with the individual needs, motivation and willingness of patients to collaborate with other healthcare providers [35]. Also, there appears to be a certain level of motivation that patients must reach before they are willing to participate in SP, highlighting the importance of finding ways to support patients in overcoming both the initial sign-up and the subsequent visits barriers [36]. Another key patient-related consideration pertains to their perception of care. Some patients are used to and prefer the biomedical model of care, wherein they favor a more passive role, and refrain from active involvement in the decision-making and, by extension, in interventions addressing non-medical determinants of health [17,32,57]. Moreover, patients’ adherence to innovative interventions and autonomy in decision-making may depend on their sociocultural background [58,59]. In addition to readiness of the context, it is essential that SP programs are also adapted to the context in which they are being implemented. The level of flexibility of an intervention or its components is one of the main ingredients of a complex intervention [60]. Flexibility in intervention delivery allows for variation in how, where, and by whom interventions are delivered and received [60]. SP is an intervention that needs to be adapted to the local context, namely the needs of the individuals, the culture, the community structure, the health system and the setting of practice [61]. In the present study, elements such as the construction of tools tailored to the local context and the reliance on the existing social service organizational structure demonstrated the flexibility of SP implementation and were identified as facilitating factors. As previously reported, SP could benefit from engagement from various healthcare professionals and macro-level approaches that establish well-defined strategic, tactical, and operational planning within the health system [32]. Hence, an absence of endorsed national policies and guidance from healthcare providers involved can pose an obstacle to active participation in SP initiatives [45].

As the implementation of a new intervention can be affected by its context, a new intervention may also, in turn, change aspects of the context in which it is delivered [23]. In this sense, interventions can be conceptualized as temporally restricted sequences of events, novel activity settings, and technologies with the capacity to catalyze transformative changes within a system through their dynamic interaction with the contextual factors and the competencies engendered by this interaction [50]. Indeed, an intervention can be viewed as a critical occurrence within the history of a system, leading to the evolution of new structures of interaction and new shared meanings [62]. Interventions affect evolving networks of person-time-place interaction, changing relationships, displacing existing activities and redistributing and transforming resources [62]. Exploring the mechanisms through which interventions bring about change is essential to understanding how the specific intervention’s effects occurred and how these effects might be replicated in similar future interventions [23].

### Strengths and limitations

This study took place in a specific SP implementation context, which has not been studied before, providing important insights on the implementation of the SP intervention, which can be explored and used to adapt new projects in other contexts. The study participants consisted of the main stakeholders involved in the SP program implementation, which provided enriched points of view by key informants. The use of CFIR made it possible to systematize the information on the implementation process comprehensively. Also, the research team included experts in evaluation research and implementation science, using several methods, including qualitative approach, which contributed to the analysis and discussion of the results through multi-disciplinary perspectives.

Nevertheless, study limitations must be acknowledged. Patients’ perceptions of the implementation process, including barriers and facilitating factors were not explored. Determinants of patient adherence and intervention effectiveness were not investigated in detail, specifically regarding the effects of SP on health outcomes, quality of life and well-being of the patients.

## Conclusions

There is currently a boost of SP initiatives in diverse contexts worldwide, but evidence gaps still remain about the implementation process of this complex intervention, i.e. what works, how and why in order to improve project scalability and sustainability. This study assessed the implementation of a pioneer SP program, providing valuable insights on the challenges of implementation in real-world contexts, which can contribute to the development of implementation science. Findings from this study may assist to design and implement future SP projects and to develop strategies that improve acceptability among patients and stakeholders, as well as intervention’s efficacy.

The findings of this study illustrate the influence of the context’ readiness for SP throughout the development of the program, demonstrate that implementation depends on the flexibility of the intervention and reinforces that the changes caused by the intervention can potentially reach a systemic level. Future investment in human resources and the development of partnerships to strengthen community responses can help improve and sustain SP interventions. Our findings suggest that it is imperative to develop strategies to promote patient health literacy, motivation, and confidence in SP and to manage their own health and well-being. Factors influencing patient adherence should be explored in future studies and may be used to guide tailored implementation improvements and delivery of SP interventions elsewhere.
